# Supplementary material for: Biased echoes: Large language models reinforce investment biases and increase portfolio risks of private investors
Source: PLoS One. 2025 Jun 27;20(6):e0325459. doi: 10.1371/journal.pone.0325459 (PMC12204588; doi:10.1371/journal.pone.0325459)
Supplement: S1 Appendix — (DOCX) [file pone.0325459.s001.docx]

# Portfolio investment risk constrasts

## Study 1

### Table A. Investment risks by LLM and relative to the benchmark (Study 1).

|  | **LLM** | **Mean** | **SD** | ***TukeyHSD p*’s** | ***Benchmark Index*** | | |
| --- | --- | --- | --- | --- | --- | --- | --- |
|  |  |  |  |  | **Value** | ***t*** | ***p*** |
| Geographical Cluster Risk | ChatGPT | 94.88 | 7.46 | *p_GPT-Copilot_* = .98 *p_GPT-Gemini_ =* .21 *p_Copilot-Gemini_ =* .15 | 58.96 | t(89) = 45.71 | < .001 |
|  | Copilot | 95.14 | 8.42 |  |  | t(88) = 40.52 | < .001 |
|  | Gemini | 92.49 | 11.95 |  |  | t(89) = 26.62 | <.001 |
| Sector Cluster Risk | ChatGPT | .30 | .11 | *p_GPT-Copilot_* = .02 *p_GPT-Gemini_ =* .74 *p_Copilot-Gemini_ =* .12 | .15 | t(89) = 12.30 | <.001 |
|  | Copilot | .36 | .22 |  |  | t(88) = 8.94 | <.001 |
|  | Gemini | .31 | .14 |  |  | t(89) = 11.14 | <.001 |
| Trend Chasing Risk | ChatGPT | 24.92 | 20.61 | *p_GPT-Copilot_* = .11 *p_GPT-Gemini_ =* .69 *p_Copilot-Gemini_ =* .01 | 9 | t(89) = 7.33 | <.001 |
|  | Copilot | 17.61 | 17.79 |  |  | t(89) = 4.59 | <.001 |
|  | Gemini | 27.92 | 32.23 |  |  | t(89) = 5.57 | <.001 |
| Active Investment Allocation Risk | ChatGPT | 57.41 | 29.21 | *p_GPT-Copilot_* = .52 *p_GPT-Gemini_ =* 1 *p_Copilot-Gemini_ =* .52 | 0 | t(89) = 18.65 | <.001 |
|  | Copilot | 51.85 | 36.62 |  |  | t(89) = 13.43 | <.001 |
|  | Gemini | 57.47 | 36.55 |  |  | t(89) = 14.91 | <.001 |
| Total Expense Risk | ChatGPT | .20 | .21 | *p_GPT-Copilot_* = .07 *p_GPT-Gemini_ =* .30  *p_Copilot-Gemini_ =* .70 | .07 | t(85) = 5.76 | <.001 |
|  | Copilot | .13 | .18 |  |  | t(67) = 2.63 | .01 |
|  | Gemini | .15 | .23 |  |  | t(87) = 3.39 | <.01 |

### Table B. Portfolio investment risks contrasts by risk-taking propensity and age (Study 1).

|  | | | **ChatGPT** | | | **Copilot** | | | **Gemini** | | |
| --- | --- | --- | --- | --- | --- | --- | --- | --- | --- | --- | --- |
|  | | | **Mean** | **SD** | ***p*’s** | **Mean** | **SD** | ***p*’s** | **Mean** | **SD** | ***p*’s** |
| Geographical Cluster Risk | Risk-taking | Low | 94.72 | 6.94 | *p_low-medium_*=1  *p_low-high_*=1  *p_medium-high_*=.89 | 95.14 | 7.42 | *p_low-medium_*=1  *p_low-high_*=1  *p_medium-high_*=1 | 90.04 | 9.47 | ***p_low-medium_*=.02**  *p_low-high_*=1  ***p_medium-high_*<.01** |
|  |  | Medium | 96.66 | 6.98 |  | 95.45 | 4.94 |  | 98.41 | 2.00 |  |
|  |  | High | 93.27 | 8.23 |  | 94.83 | 11.73 |  | 89.02 | 17.02 |  |
|  | Age | 15 | 96.11 | 5.70 | *p_15-30_*=.93  *p_15-50_* =1  *p_30-50_*=.99 | 91.63 | 10.55 | *p_15-30_*=.09  *p_15-50_*=.72  *p_30-50_*=.97 | 97.15 | 3.15 | *p_15-30_* =1  ***p_15-50_*** **<.001**  ***p_30-50_*<.001** |
|  |  | 30 | 93.20 | 9.70 |  | 98.20 | 2.00 |  | 96.25 | 4.88 |  |
|  |  | 50 | 95.35 | 6.27 |  | 95.60 | 8.96 |  | 84.07 | 17.15 |  |
| Sector Cluster Risk | Risk-taking | Low | 0.20 | 0.06 | *p_low-medium_*= .05  ***p_low-high_*<.001**  *p_medium-high_*=.55 | 0.42 | 0.33 | ***p_low-medium_*<.001**  *p_low-high_*=1  ***p_medium-high_*<.001** | 0.16 | 0.05 | ***p_low-medium_*<.001**  ***p_low-high_*<.001**  *p_medium-high_*=.85 |
|  |  | Medium | 0.31 | 0.10 |  | 0.23 | 0.08 |  | 0.41 | 0.10 |  |
|  |  | High | 0.38 | 0.09 |  | 0.44 | 0.10 |  | 0.36 | 0.09 |  |
|  | Age | 15 | 0.31 | 0.10 | *p_15-30_*=1  *p_15-50_* =1  *p_30-50_*=1 | 0.31 | 0.10 | *p_15-30_*=.28  *p_15-50_* =.89  *p_30-50_*=.99 | 0.37 | 0.15 | *p_15-30_*=.93  *p_15-50_* =.22  *p_30-50_*=.95 |
|  |  | 30 | 0.29 | 0.12 |  | 0.41 | 0.28 |  | 0.31 | 0.12 |  |
|  |  | 50 | 0.29 | 0.11 |  | 0.37 | 0.24 |  | 0.26 | 0.12 |  |
| Trend Chasing Risk | Risk-taking | Low | 2.19 | 6.96 | ***p_low-medium_*<.001**  ***p_low-high_*<.001**  *p_medium-high_*=1 | 0.67 | 3.65 | ***p_low-medium_*<.01**  ***p_low-high_*<.001**  ***p_medium-high_*<.001** | 0.00 | 0.00 | ***p_low-medium_*<.001**  ***p_low-high_*<.001**  ***p_medium-high_*<.001** |
|  |  | Medium | 36.67 | 16.73 |  | 14.00 | 10.29 |  | 63.43 | 22.61 |  |
|  |  | High | 35.90 | 13.18 |  | 38.18 | 10.19 |  | 20.31 | 22.50 |  |
|  | Age | 15 | 29.76 | 22.82 | *p_15-30_*=.95  *p_15-50_* =.97  *p_30-50_*=1 | 15.25 | 14.36 | *p_15-30_*=1  *p_15-50_* =1  *p_30-50_*=1 | 37.85 | 32.07 | *p_15-30_*=.78  ***p_15-50_* =.046**  *p_30-50_*=.85 |
|  |  | 30 | 22.18 | 17.38 |  | 20.30 | 20.09 |  | 27.65 | 34.61 |  |
|  |  | 50 | 22.83 | 21.08 |  | 17.30 | 18.65 |  | 18.25 | 27.63 |  |
| Active Investment Allocation Risk | Risk-taking | Low | 28.17 | 19.55 | ***p_low-medium_*<.001**  ***p_low-high_*<.001**  ***p_medium-high_*<.01** | 34.84 | 25.98 | *p_low-medium_*=.60  ***p_low-high_*<.001**  ***p_medium-high_*<.001** | 14.69 | 18.98 | ***p_low-medium_*<.001**  ***p_low-high_*<.001**  ***p_medium-high_*<.01** |
|  |  | Medium | 61.69 | 20.63 |  | 25.37 | 16.80 |  | 70.08 | 24.14 |  |
|  |  | High | 82.38 | 16.19 |  | 95.33 | 13.32 |  | 87.63 | 12.63 |  |
|  | Age | 15 | 62.43 | 28.18 | *p_15-30_*=.97  *p_15-50_* =1  *p_30-50_*=1 | 56.81 | 27.08 | *p_15-30_*=.99  *p_15-50_* =1  *p_30-50_*=1 | 63.54 | 38.36 | *p_15-30_*=1  *p_15-50_* =.79  *p_30-50_*=.95 |
|  |  | 30 | 52.67 | 31.43 |  | 48.50 | 39.92 |  | 59.79 | 35.35 |  |
|  |  | 50 | 57.14 | 28.06 |  | 50.23 | 41.82 |  | 49.06 | 35.54 |  |
| Total Expense Risk | Risk-taking | Low | 0.08 | 0.04 | *p_low-medium_*=.86  ***p_low-high_*<.001**  ***p_medium-high_*<.001** | 0.08 | 0.05 | *p_low-medium_*=1  ***p_low-high_*<.001**  ***p_medium-high_*<.001** | 0.03 | 0.01 | *p_low-medium_*=1  ***p_low-high_*<.001**  ***p_medium-high_*<.001** |
|  |  | Medium | 0.14 | 0.10 |  | 0.06 | 0.04 |  | 0.05 | 0.03 |  |
|  |  | High | 0.41 | 0.26 |  | 0.51 | 0.24 |  | 0.39 | 0.29 |  |
|  | Age | 15 | 0.17 | 0.17 | *p_15-30_*=.97  *p_15-50_* =1  *p_30-50_*=1 | 0.19 | 0.25 | *p_15-30_*=.67  *p_15-50_* =.58  *p_30-50_*=1 | 0.12 | 0.20 | *p_15-30_*=1  *p_15-50_* =.95  *p_30-50_*=1 |
|  |  | 30 | 0.23 | 0.21 |  | 0.08 | 0.05 |  | 0.16 | 0.25 |  |
|  |  | 50 | 0.20 | 0.24 |  | 0.07 | 0.04 |  | 0.18 | 0.25 |  |

## Study 2a

### Table C. Investment risks by LLM and relative to the benchmark (Study 2a).

|  | **Condition** | **Mean** | **SD** | ***t-test p*** | ***Benchmark Index*** | | |
| --- | --- | --- | --- | --- | --- | --- | --- |
|  |  |  |  |  | **Value** | ***t*** | ***p*** |
| Geographical Cluster Risk | Control | 94.94 | 8.89 | <.01 | 58.96 | t(89) = 38.39 | <.001 |
|  | Debiased | 90.39 | 11.69 |  |  | t(89) = 25.51 | <.001 |
| Sector Cluster Risk | Control | .32 | .13 | .04 | .15 | t(89) = 11.91 | <.001 |
|  | Debiased | .28 | .14 |  |  | t(89) = 8.36 | <.001 |
| Trend Chasing Risk | Control | 24.50 | 20.03 | .80 | 9 | t(89) = 7.34 | <.001 |
|  | Debiased | 23.64 | 24.26 |  |  | t(89) = 5.72 | <.001 |
| Active Investment Allocation Risk | Control | 59.57 | 30.99 | .04 | 0 | t(89) = 18.24 | <.001 |
|  | Debiased | 48.72 | 39.64 |  |  | t(89) = 11.66 | <.001 |
| Total Expense Risk | Control | .19 | .21 | .64 | .07 | t(85) = 5.34 | <.001 |
|  | Debiased | .18 | .20 |  |  | t(76) = 4.63 | <.001 |

### Table D. Portfolio investment risks contrasts by risk-taking propensity and age (Study 2a).

|  | | | **Control prompt** | | | **Debiased prompt** | | | ***p_control-debiased_*** |
| --- | --- | --- | --- | --- | --- | --- | --- | --- | --- |
|  | | | **Mean** | **SD** | ***p*’s** | **Mean** | **SD** | ***p*’s** |  |
| Geographical Cluster Risk | Risk-taking | Low | 96.97 | 1.46 | *p_low-medium_*=1  *p_low-high_*=.18  *p_medium-high_*=.17 | 86.89 | 11.11 | *p_low-medium_*=.60  *p_low-high_*=.15  *p_medium-high_*=.96 | **<.01** |
|  |  | Medium | 97.03 | 4.53 |  | 91.05 | 12.74 |  | .20 |
|  |  | High | 90.82 | 13.91 |  | 93.23 | 10.59 |  | .94 |
|  | Age | 15 | 95.85 | 7.90 | *p_15-30_*=.89  *p_15-50_* =1  *p_30-50_*=.88 | 93.44 | 10.21 | *p_15-30_*=.17  *p_15-50_* =.90  *p_30-50_*=.78 | .94 |
|  |  | 30 | 93.01 | 12.08 |  | 87.12 | 13.55 |  | .24 |
|  |  | 50 | 95.96 | 5.33 |  | 90.61 | 10.52 |  | .34 |
| Sector Cluster Risk | Risk-taking | Low | 0.21 | 0.11 | *p_low-medium_*<.001  *p_low-high_*<.001  *p_medium-high_*=.09 | 0.15 | 0.04 | *p_low-medium_*<.001  *p_low-high_*<.001  *p_medium-high_*<.001 | .20 |
|  |  | Medium | 0.34 | 0.12 |  | 0.27 | 0.12 |  | .10 |
|  |  | High | 0.41 | 0.09 |  | 0.41 | 0.11 |  | 1 |
|  | Age | 15 | 0.39 | 0.14 | *p_15-30_*=02  *p_15-50_* =.13  *p_30-50_*=.98 | 0.30 | 0.13 | *p_15-30_*=.93  *p_15-50_* =.90  *p_30-50_*=1 | .14 |
|  |  | 30 | 0.27 | 0.12 |  | 0.27 | 0.14 |  | 1 |
|  |  | 50 | 0.30 | 0.12 |  | 0.26 | 0.15 |  | .91 |
| Trend Chasing Risk | Risk-taking | Low | 5.81 | 13.36 | *p_low-medium_*<.001  *p_low-high_*<.001  *p_medium-high_*=.93 | 0.49 | 2.67 | *p_low-medium_*<.001  *p_low-high_*<.001  *p_medium-high_*=.06 | .80 |
|  |  | Medium | 35.84 | 17.84 |  | 29.36 | 25.27 |  | .64 |
|  |  | High | 31.85 | 13.59 |  | 41.08 | 16.07 |  | .24 |
|  | Age | 15 | 29.33 | 17.05 | *p_15-30_*=.67  *p_15-50_* =.90  *p_30-50_*=1 | 31.20 | 26.20 | *p_15-30_*=.60  *p_15-50_* =.17  *p_30-50_*=.97 | 1 |
|  |  | 30 | 20.84 | 20.96 |  | 22.08 | 23.32 |  | 1 |
|  |  | 50 | 23.32 | 21.50 |  | 17.64 | 21.85 |  | .92 |
| Active Investment Allocation Risk | Risk-taking | Low | 29.39 | 25.79 | *p_low-medium_*<.001  *p_low-high_*<.001  *p_medium-high_*<.01 | 12.09 | 13.48 | *p_low-medium_*<.001  *p_low-high_*<.001  *p_medium-high_*<.001 | .**049** |
|  |  | Medium | 63.22 | 20.70 |  | 47.64 | 40.08 |  | .10 |
|  |  | High | 86.09 | 13.03 |  | 86.44 | 13.17 |  | .35 |
|  | Age | 15 | 70.12 | 28.63 | *p_15-30_*=.30  *p_15-50_* =.69  *p_30-50_*=.99 | 63.73 | 38.05 | *p_15-30_*=.22  *p_15-50_* =.07  *p_30-50_*=1 | .98 |
|  |  | 30 | 51.49 | 33.95 |  | 43.47 | 38.69 |  | .95 |
|  |  | 50 | 57.08 | 28.02 |  | 38.97 | 38.96 |  | .33 |
| Total Expense Risk | Risk-taking | Low | 0.07 | 0.04 | *p_low-medium_*=.98  *p_low-high_*<.001  *p_medium-high_*<.001 | 0.07 | 0.03 | *p_low-medium_*=.94  *p_low-high_*<.001  *p_medium-high_*<.001 | 1 |
|  |  | Medium | 0.10 | 0.06 |  | 0.11 | 0.06 |  | .97 |
|  |  | High | 0.43 | 0.24 |  | 0.40 | 0.26 |  | .97 |
|  | Age | 15 | 0.21 | 0.24 | *p_15-30_*=.99  *p_15-50_* =1  *p_30-50_*=1 | 0.19 | 0.23 | *p_15-30_*=1  *p_15-50_* =1  *p_30-50_*=1 | 1 |
|  |  | 30 | 0.18 | 0.21 |  | 0.18 | 0.22 |  | 1 |
|  |  | 50 | 0.19 | 0.19 |  | 0.16 | 0.15 |  | .99 |

## Study 2b

### Table E. Investment risks by LLM and relative to the benchmark (Study 2b).

|  | **Condition** | **Mean** | **SD** | ***t-test p*** | ***Benchmark Index*** | | |
| --- | --- | --- | --- | --- | --- | --- | --- |
|  |  |  |  |  | **Value** | ***t*** | ***p*** |
| Geographical Cluster Risk | Control | 94.52 | 8.61 | <.001 | 58.96 | t(89) = 39.17 | <.001 |
|  | Debiased | 84.06 | 12.71 |  |  | t(89) = 18.74 | <.001 |
| Sector Cluster Risk | Control | .32 | .14 | <.001 | .15 | t(89) = 11.3 | <.001 |
|  | Debiased | .20 | .10 |  |  | t(89) = 4.12 | <.001 |
| Trend Chasing Risk | Control | 25.87 | 20.89 | <.001 | 9 | t(89) = 7.66 | <.001 |
|  | Debiased | 8.84 | 14.51 |  |  | t(89) = -.11 | **.91** |
| Active Investment Allocation Risk | Control | 58.12 | 31.25 | <.001 | 0 | t(89) = 17.64 | <.001 |
|  | Debiased | 25.19 | 28.43 |  |  | t(89) = 8.41 | <.001 |
| Total Expense Risk | Control | .20 | .21 | .02 | .07 | t(83) = 5.74 | <.001 |
|  | Debiased | .14 | .14 |  |  | t(89) = 4.52 | <.001 |

### Table F. Portfolio investment risks contrasts by risk-taking propensity and age (Study 2b).

|  | | | **Control prompt** | | | **Debiased prompt** | | | **p_control-debiased_** |
| --- | --- | --- | --- | --- | --- | --- | --- | --- | --- |
|  | | | **Mean** | **SD** | ***p*’s** | **Mean** | **SD** | ***p*’s** |  |
| Geographical Cluster Risk | Risk-taking | Low | 95.91 | 4.41 | *p_low-medium_*=.99  *p_low-high_*=.25  *p_medium-high_*=.06 | 89.55 | 9.13 | *p_low-medium_*<.001  *p_low-high_*=.42  *p_medium-high_*=.13 | .16 |
|  |  | Medium | 97.55 | 3.70 |  | 78.01 | 13.51 |  | **<.001** |
|  |  | High | 90.14 | 12.78 |  | 84.62 | 12.65 |  | .29 |
|  | Age | 15 | 96.02 | 6.77 | *p_15-30_*=.97  *p_15-50_* =.96  *p_30-50_*=1 | 87.35 | 13.03 | *p_15-30_*=.22  *p_15-50_* =.78  *p_30-50_*=.94 | .03 |
|  |  | 30 | 93.90 | 8.26 |  | 81.11 | 13.64 |  | **<.001** |
|  |  | 50 | 93.68 | 10.50 |  | 83.72 | 10.93 |  | **<.01** |
| Sector Cluster Risk | Risk-taking | Low | 0.20 | 0.08 | *p_low-medium_*<.001  *p_low-high_*<.001  *p_medium-high_*=.04 | 0.15 | 0.02 | *p_low-medium_*=.03  *p_low-high_*=.12  *p_medium-high_*<.01 | .41 |
|  |  | Medium | 0.35 | 0.11 |  | 0.18 | 0.09 |  | **<.001** |
|  |  | High | 0.42 | 0.12 |  | 0.26 | 0.12 |  | **<.001** |
|  | Age | 15 | 0.36 | 0.13 | *p_15-30_*=.42  *p_15-50_* =.51  *p_30-50_*=1 | 0.22 | 0.12 | *p_15-30_*=.93  *p_15-50_* =.70  *p_30-50_*=1 | **<.001** |
|  |  | 30 | 0.30 | 0.13 |  | 0.19 | 0.10 |  | **<.01** |
|  |  | 50 | 0.30 | 0.16 |  | 0.18 | 0.08 |  | **<.01** |
| Trend Chasing Risk | Risk-taking | Low | 3.26 | 8.87 | *p_low-medium_*<.001  *p_low-high_*<.001  *p_medium-high_*=1 | 0.00 | 0.00 | *p_low-medium_*=.18  *p_low-high_*<.001  *p_medium-high_*=.02 | .93 |
|  |  | Medium | 37.12 | 17.09 |  | 7.88 | 11.68 |  | **<.001** |
|  |  | High | 37.21 | 13.25 |  | 18.63 | 18.12 |  | **<.001** |
|  | Age | 15 | 29.13 | 19.10 | *p_15-30_*=.91  *p_15-50_* =.88  *p_30-50_*=1 | 12.49 | 15.95 | *p_15-30_*=.96  *p_15-50_* =.65  *p_30-50_*=.98 | **<.01** |
|  |  | 30 | 24.45 | 20.98 |  | 8.63 | 14.48 |  | **.01** |
|  |  | 50 | 24.01 | 22.74 |  | 5.39 | 12.48 |  | **<.01** |
| Active Investment Allocation Risk | Risk-taking | Low | 26.40 | 20.99 | *p_low-medium_*<.001  *p_low-high_*<.001  *p_medium-high_*=.02 | 13.15 | 15.11 | *p_low-medium_*=1  *p_low-high_*<.001  *p_medium-high_*<.001 | .19 |
|  |  | Medium | 64.65 | 22.76 |  | 14.54 | 18.39 |  | **<.001** |
|  |  | High | 83.30 | 17.26 |  | 47.89 | 33.37 |  | **<.001** |
|  | Age | 15 | 65.06 | 28.17 | *p_15-30_*=.93  *p_15-50_* =.50  *p_30-50_*=.97 | 32.61 | 29.28 | *p_15-30_*=.59  *p_15-50_* =.79  *p_30-50_*=1 | **<.001** |
|  |  | 30 | 57.73 | 34.11 |  | 20.28 | 29.29 |  | **<.001** |
|  |  | 50 | 51.57 | 30.76 |  | 22.70 | 26.01 |  | **<.01** |
| Total Expense Risk | Risk-taking | Low | 0.07 | 0.03 | *p_low-medium_*=.57  *p_low-high_*=<.001  *p_medium-high_*<.001 | 0.08 | 0.04 | *p_low-medium_*=.99  *p_low-high_*=<.001  *p_medium-high_*<.01 | 1 |
|  |  | Medium | 0.13 | 0.08 |  | 0.10 | 0.05 |  | .90 |
|  |  | High | 0.42 | 0.25 |  | 0.24 | 0.20 |  | **<.001** |
|  | Age | 15 | 0.20 | 0.23 | *p_15-30_*=1  *p_15-50_* =.99  *p_30-50_*=.91 | 0.16 | 0.18 | *p_15-30_*=.98  *p_15-50_* =.98  *p_30-50_*=1 | .93 |
|  |  | 30 | 0.23 | 0.23 |  | 0.13 | 0.10 |  | .27 |
|  |  | 50 | 0.18 | 0.18 |  | 0.13 | 0.13 |  | .89 |

## Study 3

### Table G. Investment risks by LLM and relative to the benchmark (Study 3).

|  | **Condition** | **Mean** | **SD** | ***t-test p*** | ***Benchmark Index*** | | |
| --- | --- | --- | --- | --- | --- | --- | --- |
|  |  |  |  |  | **Value** | ***t*** | ***p*** |
| Geographical Cluster Risk | Control | 95.18 | 7.10 | <.001 | 58.96 | t(89) = 48.4 | <.001 |
|  | Goal | 89.04 | 13.44 |  |  | t(89) = 21.23 | <.001 |
| Sector Cluster Risk | Control | .33 | .15 | .83 | .15 | t(89) = 11.72 | <.001 |
|  | Goal | .34 | .19 |  |  | t(89) = 9.1 | <.001 |
| Trend Chasing Risk | Control | 26.95 | 22.46 | <.001 | 9 | t(89) = 7.58 | <.001 |
|  | Goal | 7.63 | 10.53 |  |  | t(89) = -1.23 | **.22** |
| Active Investment Allocation Risk | Control | 59.41 | 30.45 | <.001 | 0 | t(89) = 18.51 | <.001 |
|  | Goal | 88.51 | 17.39 |  |  | t(89) = 48.29 | <.001 |
| Total Expense Risk | Control | .18 | .20 | <.001 | .07 | t(80) = 5.23 | <.001 |
|  | Goal | .32 | .14 |  |  | t(88) = 17.32 | <.001 |
| ESG Score | Control | 71.59 | 6.60 | .98 | 71.12 | t(89) = .67 | **.50** |
|  | Goal | 71.61 | 4.25 |  |  | t(89) = 1.08 | **.28** |

### Table H. Portfolio investment risks contrasts by risk-taking propensity and age (Study 3).

|  | | | **Control prompt** | | | **Goal prompt** | | | **p_control-goal_** |
| --- | --- | --- | --- | --- | --- | --- | --- | --- | --- |
|  | | | **Mean** | **SD** | ***p*’s** | **Mean** | **SD** | ***p*’s** |  |
| Geographical Cluster Risk | Risk-taking | Low | 96.62 | 2.31 | *p_low-medium_*=.99  *p_low-high_*=.93  *p_medium-high_*=1 | 89.20 | 13.39 | *p_low-medium_*=.97  *p_low-high_*=.99  *p_medium-high_*=.72 | .09 |
|  |  | Medium | 94.98 | 8.16 |  | 87.01 | 13.88 |  | .05 |
|  |  | High | 93.92 | 8.88 |  | 90.92 | 13.22 |  | .89 |
|  | Age | 15 | 96.37 | 5.96 | *p_15-30_*=1  *p_15-50_* =94  *p_30-50_*=.99 | 92.36 | 11.21 | *p_15-30_*=.85  *p_15-50_* =.15  *p_30-50_*=.80 | .69 |
|  |  | 30 | 95.36 | 7.26 |  | 89.14 | 12.02 |  | .22 |
|  |  | 50 | 93.79 | 7.93 |  | 85.64 | 16.15 |  | **.04** |
| Sector Cluster Risk | Risk-taking | Low | 0.23 | 0.12 | *p_low-medium_*=.17  *p_low-high_*<.001  *p_medium-high_*=.10 | 0.28 | 0.20 | *p_low-medium_*=.59  *p_low-high_*=.10  *p_medium-high_*=.92 | .86 |
|  |  | Medium | 0.33 | 0.10 |  | 0.35 | 0.16 |  | 1 |
|  |  | High | 0.44 | 0.14 |  | 0.39 | 0.20 |  | .86 |
|  | Age | 15 | 0.37 | 0.13 | *p_15-30_*=.98  *p_15-50_* =.38  *p_30-50_*=.82 | 0.32 | 0.12 | *p_15-30_*=.96  *p_15-50_* =.99  *p_30-50_*=1 | .81 |
|  |  | 30 | 0.34 | 0.17 |  | 0.35 | 0.21 |  | 1 |
|  |  | 50 | 0.29 | 0.13 |  | 0.34 | 0.24 |  | .79 |
| Trend Chasing Risk | Risk-taking | Low | 6.0 | 12.48 | *p_low-medium_*<.001  *p_low-high_*<.001  *p_medium-high_*=.75 | 0.5 | 2.01 | *p_low-medium_*=.03  *p_low-high_*=.03  *p_medium-high_*=1 | .63 |
|  |  | Medium | 39.80 | 19.54 |  | 11.12 | 11.05 |  | **<.001** |
|  |  | High | 35.04 | 17.83 |  | 11.28 | 11.62 |  | **<.001** |
|  | Age | 15 | 32.40 | 20.54 | *p_15-30_*=.91  *p_15-50_* =.10  *p_30-50_*=.60 | 9.49 | 12.57 | *p_15-30_*=.97  *p_15-50_* =1  *p_30-50_*=1 | **<.001** |
|  |  | 30 | 27.81 | 23.36 |  | 5.97 | 8.88 |  | **<.001** |
|  |  | 50 | 20.62 | 22.53 |  | 7.45 | 9.85 |  | **.04** |
| Active Investment Allocation Risk | Risk-taking | Low | 33.11 | 20.42 | *p_low-medium_*<.001  *p_low-high_*<.001  *p_medium-high_*<.001 | 89.65 | 21.19 | *p_low-medium_*=1  *p_low-high_*=1  *p_medium-high_*=1 | **<.001** |
|  |  | Medium | 60.83 | 25.12 |  | 87.50 | 13.15 |  | **<.001** |
|  |  | High | 84.29 | 20.99 |  | 88.37 | 17.42 |  | .97 |
|  | Age | 15 | 66.37 | 25.05 | *p_15-30_*=.94  *p_15-50_* =.18  *p_30-50_*=.71 | 90.84 | 14.79 | *p_15-30_*=1  *p_15-50_* =.94  *p_30-50_*=.97 | **<.01** |
|  |  | 30 | 60.49 | 33.05 |  | 89.78 | 17.34 |  | **<.001** |
|  |  | 50 | 51.37 | 31.75 |  | 84.89 | 19.68 |  | **<.001** |
| Total Expense Risk | Risk-taking | Low | 0.08 | 0.08 | *p_low-medium_*=.48  *p_low-high_*<.001  *p_medium-high_*<.001 | 0.32 | 0.13 | *p_low-medium_*=1  *p_low-high_*=1  *p_medium-high_*=1 | **<.001** |
|  |  | Medium | 0.15 | 0.15 |  | 0.32 | 0.11 |  | **<.001** |
|  |  | High | 0.37 | 0.24 |  | 0.32 | 0.16 |  | .78 |
|  | Age | 15 | 0.19 | 0.22 | *p_15-30_*=.97  *p_15-50_* =1  *p_30-50_*=.77 | 0.29 | 0.12 | *p_15-30_*=1  *p_15-50_* =.84  *p_30-50_*=.98 | .17 |
|  |  | 30 | 0.15 | 0.16 |  | 0.31 | 0.13 |  | **<.01** |
|  |  | 50 | 0.21 | 0.20 |  | 0.35 | 0.15 |  | **<.01** |
| ESG Score | Risk-taking | Low | 73.73 | 4.22 | *p_low-medium_*=.71  *p_low-high_*<.001  *p_medium-high_*<.001 | 72.50 | 3.08 | *p_low-medium_*=1  *p_low-high_*=.40  *p_medium-high_*=.61 | .91 |
|  |  | Medium | 75.44 | 3.32 |  | 72.11 | 4.04 |  | .07 |
|  |  | High | 65.60 | 6.90 |  | 70.21 | 5.16 |  | **<.01** |
|  | Age | 15 | 72.63 | 7.27 | *p_15-30_*=  *p_15-50_* =  *p_30-50_*= | 72.89 | 3.93 | *p_15-30_*=  *p_15-50_* =  *p_30-50_*= | 1 |
|  |  | 30 | 71.93 | 6.00 |  | 71.37 | 3.99 |  | 1 |
|  |  | 50 | 70.20 | 6.44 |  | 70.56 | 4.62 |  | 1 |

## Robustness study

### Table I. Investment risks by LLM and relative to the benchmark (Robustness study).

|  | **LLM** | **Mean** | **SD** | ***TukeyHSD p*’s** | ***Benchmark Index*** | | |
| --- | --- | --- | --- | --- | --- | --- | --- |
|  |  |  |  |  | **Value** | ***t*** | ***p*** |
| Geographical Cluster Risk | LLMs Study 1 | 94.17 | 9.52 | *p_GPT-S1_* = .43 *p_GPT-Sonnet_ =* .97 *p_S1-Sonnet_ =* .62 | 58.96 | t(268) =60.66 | <.001 |
|  | GPT 4o | 96.03 | 8.42 |  |  | t(44) =29.52 | <.001 |
|  | Sonnet 3.5 | 95.57 | 8.80 |  |  | t(44) = 27.90 | <.001 |
| Sector Cluster Risk | LLMs Study 1 | .32 | .16 | ***p_GPT-S1_* <.001 *p_GPT-Sonnet_* <.01 *p_S1-Sonnet_* <.001** | .15 | t(268) = 17.03 | <.001 |
|  | GPT 4o | .54 | .21 |  |  | t(44) = 12.52 | <.001 |
|  | Sonnet 3.5 | .69 | .33 |  |  | t(44) = 10.92 | <.001 |
| Trend Chasing Risk | LLMs Study 1 | 23.48 | 24.65 | *p_GPT-S1_* = .65 *p_GPT-Sonnet_ =* .10 *p_S1-Sonnet_ =* .16 | 9 | t(269) = 9.65 | <.001 |
|  | GPT 4o | 26.80 | 20.76 |  |  | t(44) = 5.75 | <.001 |
|  | Sonnet 3.5 | 16.66 | 14.05 |  |  | t(44) = 3.66 | <.001 |
| Active Investment Allocation Risk | LLMs Study 1 | 55.50 | 34.20 | *p_GPT-S1_* = .99 *p_GPT-Sonnet_ =* .11 ***p_S1-Sonnet_ =* .03** | 0 | t(269) = 26.66 | <.001 |
|  | GPT 4o | 56.22 | 42.31 |  |  | t(44) = 8.91 | <.001 |
|  | Sonnet 3.5 | 41.07 | 34.12 |  |  | t(44) = 8.08 | <.001 |
| Total Expense Risk | LLMs Study 1 | .16 | .21 | ***p_GPT-S1_* <.01** *p_GPT-Sonnet_ =* .69  ***p_S1-Sonnet_ =* .03** | .07 | t(241) = 6.83 | <.001 |
|  | GPT 4o | .30 | .32 |  |  | t(44) = 4.88 | <.001 |
|  | Sonnet 3.5 | .26 | .20 |  |  | t(44) = 4.25 | <.001 |
