# Supplementary material for: Biased echoes: Large language models reinforce investment biases and increase portfolio risks of private investors
Source: PLoS One. 2025 Jun 27;20(6):e0325459. doi: 10.1371/journal.pone.0325459 (PMC12204588; doi:10.1371/journal.pone.0325459)

# Extended concentration analysis

We conducted an extended concentration analysis to gain deeper understanding into the geographical regions and sectors that are overrepresented in LLM-generated financial investment advice relative to the benchmark across all studies.

## Study 1

### Fig A. Robust geographical and sector concentration across LLMs (Study 1).

###
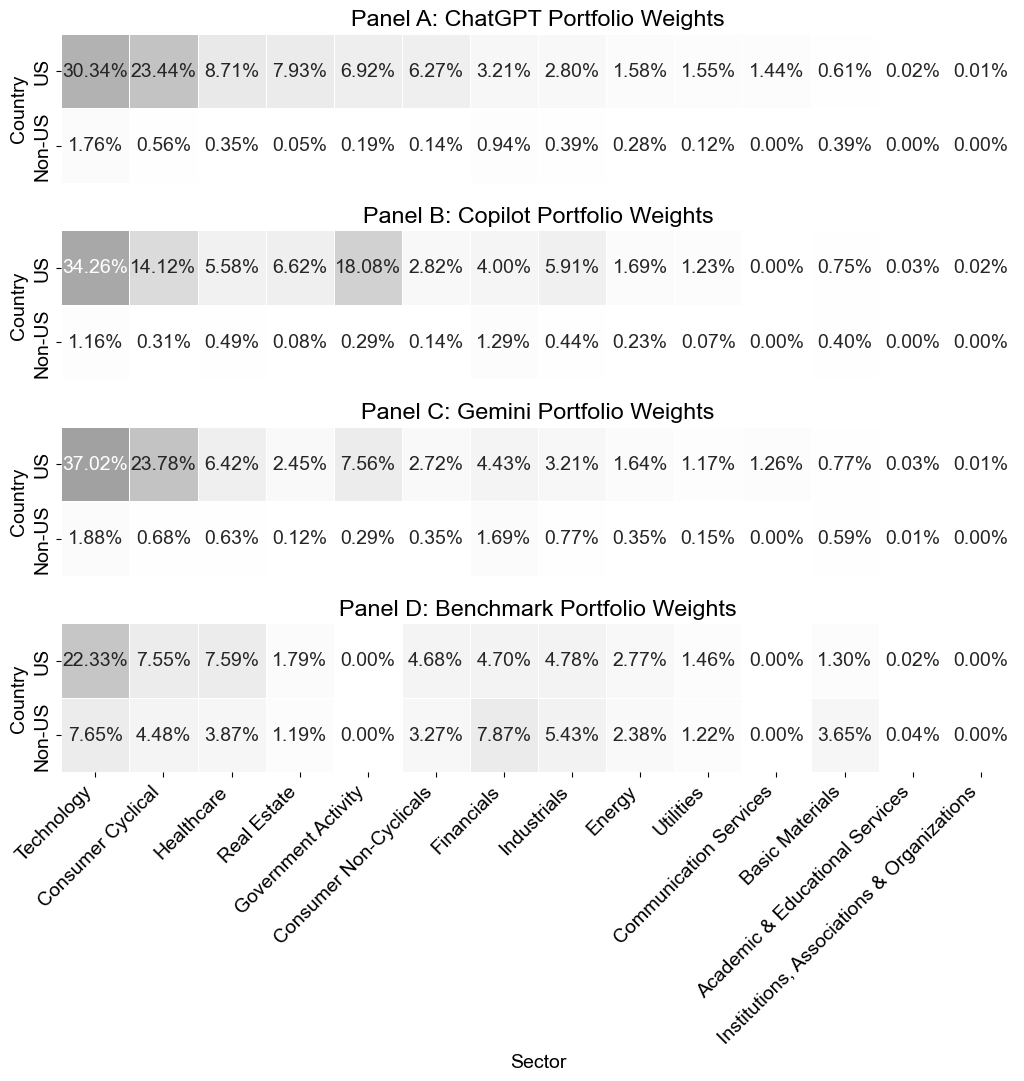


## Study 2a

### Fig A. Narrow debiasing interventions have a small effect on the geographical and sector concentration (Study 2a).

###
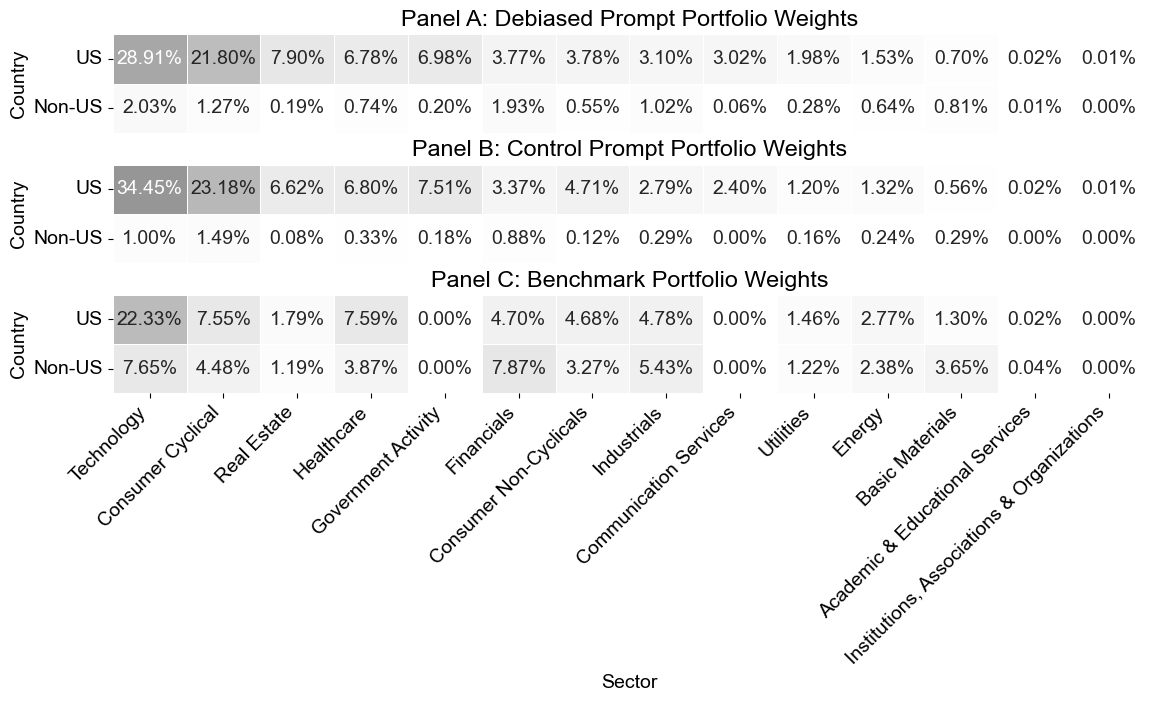


## Study 2b

### Fig A. Broad debiasing interventions have a moderate effect on the geographical and sector concentration (Study 2b).

###
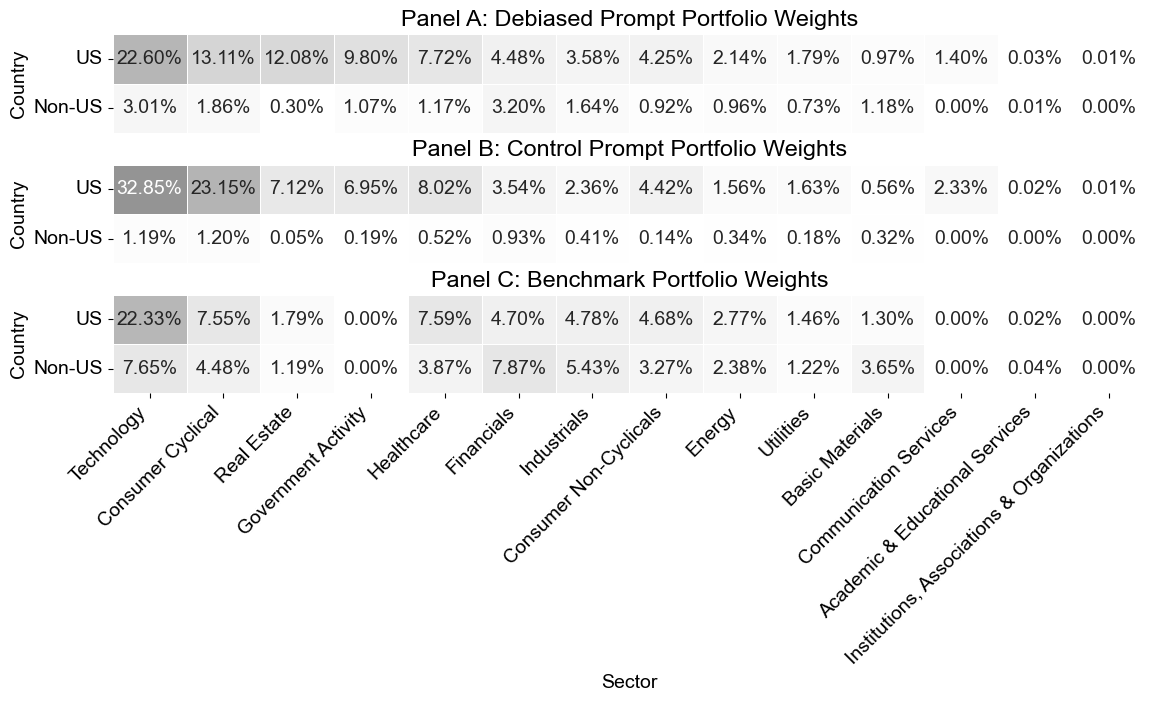


## Study 3

### Fig A. Social responsibility goal formulations shift the sector concentration (Study 3).

###
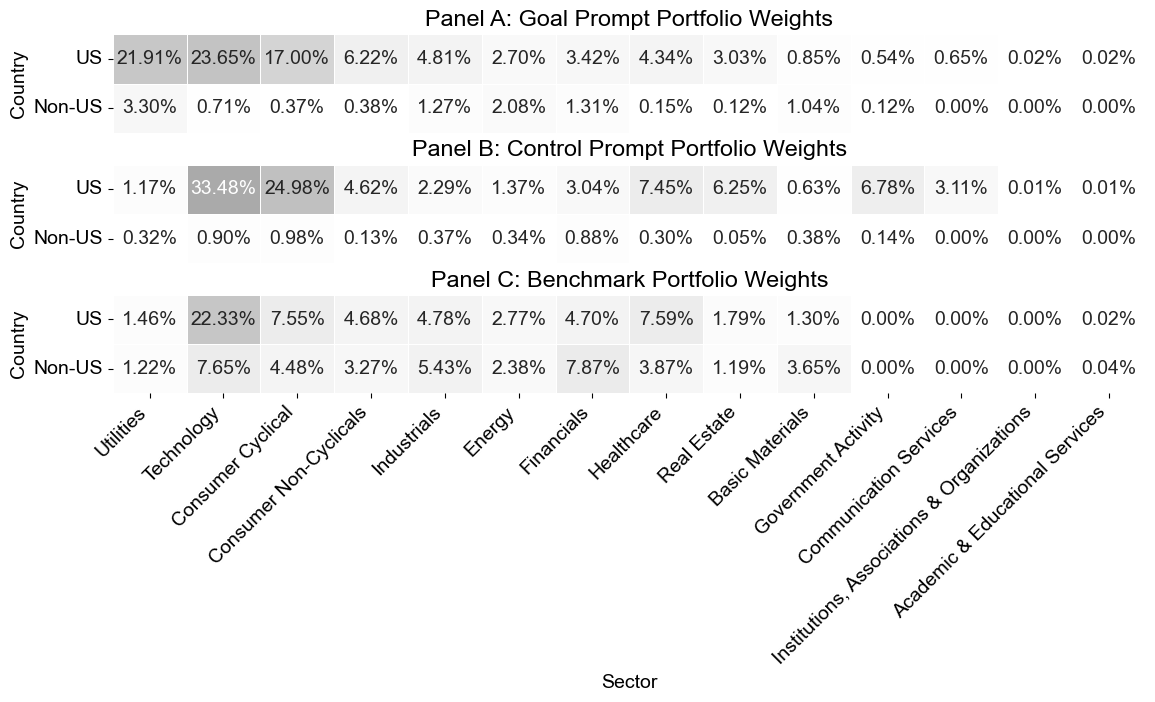

Supplement: S2 Appendix — (DOCX) [file pone.0325459.s002.docx]
