# Supplementary material for: Biased echoes: Large language models reinforce investment biases and increase portfolio risks of private investors
Source: PLoS One. 2025 Jun 27;20(6):e0325459. doi: 10.1371/journal.pone.0325459 (PMC12204588; doi:10.1371/journal.pone.0325459)
Supplement: S3 Appendix — (DOCX) [file pone.0325459.s003.docx]

# LLM financial advice language style analysis

### Table A. Across all studies we find a high presence of disclaimers, rationale, and assertiveness

|  | Presence of Disclaimers | Rationale | Assertiveness |
| --- | --- | --- | --- |
| Study 1 | .9264 | .9623 | .5924 |
| Study 2a | .9521 | .9560 | .6060 |
| Study 2b | .9462 | .9602 | .6172 |
| Study 3 | .9403 | .9535 | .6299 |

## Study 1

We observe a high presence of disclaimer notes (M_disclaimer_ = .9264), offering a rationale (M_rational_ = .9623) and assertiveness (M_assertiveness_ = .5924). While all LLMs scored high across these dimensions, we find a smaller presence of disclaimers in Gemini financial advice (M_ChatGPT_ = .9467, SD_ChatGPT_ = .0525; M_Copilot_ = .9474, SD_Copilot_ = .0418; M_Gemini_ = .8850, SD_Gemini_ = .0663; *p_ChatGPT-Copilot_* > .05; all other *p* < .001), fewer rationale in ChatGPT financial advice (M_ChatGPT_ = .9548, SD_ChatGPT_ = .0246; M_Copilot_ = .9690, SD_Copilot_ = .0161; M_Gemini_ = .9632, SD_Gemini_ = .0162; *p_Copilot-Gemini_* > .05; *p_ChatGPT-Gemini_* < .05; *p_ChatGPT-Copilot_* < .001) and more assertive tone in financial advice of ChatGPT compared to Copilot (M_ChatGPT_ = .6102, SD_ChatGPT_ = .1067; M_Copilot_ = .5725, SD_Copilot_ = .103; M_Gemini_ = .5945, SD_Gemini_ = .1004; *p_ChatGPT-Copilot_* < .05; all other *p* > .05). Further, we find evidence for a smaller presence of disclaimers in financial advice of LLMs for investors with low risk tendency (M_low_ = .9012, SD_low_ = .068; M_medium_ = .9343, SD_medium_ = .0627; M_high_ = .9437, SD_high_ = .0439; *p_medium-high_* > .05; all other *p* < .001) and higher levels of assertiveness for investors with high risk tendency (M_low_ = .5632, SD_low_ = .111; M_medium_ = .5688, SD_medium_ = .0988; M_high_ = .6453, SD_high_ = .0804; *p_low-medium_* > .05; all other *p* < .001).

## Study 2a

In Study 2a we also find a high presence of disclaimer notes (M_disclaimer_ = .9521), a greater presence of a rationale (M_rational_ = .956) and assertiveness (M_assertiveness_ = .606) in LLM financial advice. Further, we find smaller presence of disclaimer notes (M_Control_= .9592, SD_Control_ = .0316; M_Debiased_ = .9451, SD_Debiased_= .0438; *p* < .05) and less assertiveness (M_Control_= .6289, SD_Control_ = .124; M_Debiased_ = .5831, SD_Debiased_= .1167; *p* < .05) in the debiased prompt condition compared to the control condition but no difference in rationale (M_Control_= .9592, SD_Control_ = .0209; M_Debiased_ = .9527, SD_Debiased_= .0262; *p* > .05). We find a smaller presence of disclaimer notes in LLM financial advice for investors with low risk tendency (M_low_= .9384, SD_low_ = .0478; M_medium_= .9551, SD_medium_= .034; M_high_= .9629, SD_high_= .0282; *p_medium-high_* > .05; *p_low-high_* < .01; *p_low-medium_* < .05) and higher assertiveness for investors with high risk tendency (M_low_= .5718, SD_low_ = .11; M_medium_= .5847, SD_medium_= .12; M_high_= .6615, SD_high_= .1185; *p_low-medium_* > .05; *p_low-high_* < .001; *p_medium-high_* < .01).

## Study 2b

Corroborating the findings of Study 1, we find an overall high presence of disclaimer notes (M_disclaimer_ = .9462), offering a rationale (M_rational_ = .9602), and assertiveness. (M_assertiveness_ = .6172). We observe a smaller presence of disclaimer notes in the debiased prompt condition compared to the control condition (M_Control_= .9588, SD_Control_ = .0419; M_Debiased_ = .9336, SD_Debiased_= .0587; *p* < .01) but no differences in rationale (M_Control_= .9620, SD_Control_ = .0227; M_Debiased_ = .9583, SD_Debiased_= .0251; *p* > .05) and assertiveness (M_Control_= .6065, SD_Control_ = .1197; M_Debiased_ = .6278, SD_Debiased_= .1281; *p* > .05) across conditions. Analogous to Study 1 we find evidence for a lower presence of disclaimer notes for investors with low risk tendency (M_low_= .9237, SD_low_ = .0624; M_medium_= .9510, SD_medium_= .052; M_high_= .9638, SD_high_= .0295; *p_medium-high_* > .05; *p_low-medium_* < .01; *p_low-high_* < .001). Additionally, we observe higher levels of assertiveness for investors with more extreme risk tendencies compared to medium risk tendencies (M_low_= .6405, SD_low_ = .1246; M_medium_= .5755, SD_medium_= .1195; M_high_= .6356, SD_high_= .1193; *p_low-high_* > .05; all other *p* < .05;).

## Study 3

Again, we observe high levels of disclaimer notes (M_disclaimer_ = .9403), providing a rationale (M_rational_ = .9535) and assertiveness (M_assertiveness_ = .6299) in LLM financial advice. Again, we find smaller presence of disclaimer notes in the goal condition (i.e., prompt-engineered condition) compared to the control condition (M_Control_= .954, SD_Control_ = .044; M_Debiased_ = .9266, SD_Debiased_= .054; *p* < .001) but higher levels of assertiveness (M_Control_= .6129, SD_Control_ = .1062; M_Debiased_ = .6469, SD_Debiased_= .1068; *p* < .05). Further, corroborating the findings of all earlier studies, we find a smaller presence of disclaimer notes in LLM financial advice for investors with low risk tendency (M_low_= .9202, SD_low_ = .0594; M_medium_= .9457, SD_medium_= .0418; M_high_= .955, SD_high_= .0441; *p_medium-high_* > .05; *p_low-high_* < .001; *p_low-medium_* < .05).
