# Supplementary material for: Biased echoes: Large language models reinforce investment biases and increase portfolio risks of private investors
Source: PLoS One. 2025 Jun 27;20(6):e0325459. doi: 10.1371/journal.pone.0325459 (PMC12204588; doi:10.1371/journal.pone.0325459)
Supplement: S4 Appendix — (DOCX) [file pone.0325459.s004.docx]

# Exclusion criteria and robustness checks

To compute the investment risk measures, we utilized the Yahoo Finance and Refinitiv Eikon databases. However, these databases occasionally fail to provide complete geolocation, sector, or TER data for certain positions in the LLM financial advice or their holdings. In such instances, we exclude these positions from our analysis. Moreover, there are cases where LLMs might offer financial advice that does not include any investment positions (i.e., resolvable ticker symbols), but rather savings products (e.g., savings accounts or defined contributions plans). These portfolios and positions are also excluded from our analysis due to data unavailability. For a comprehensive list of portfolio position exclusion criteria refer to Table A.

### Table A. (Sub-)Position exclusion criteria by investment risk.

| Measure | (Sub-)Position exclusion Criteria |
| --- | --- |
| Geographical cluster risk | Excludes positions if geolocation is unknown or if it’s a savings product. |
| Sector cluster risk | Excludes positions if sector is unknown or if it’s a savings product. |
| Trend chasing risk | - |
| Active investment allocation risk | Excludes savings products. Active investments include all investments that do not replicate a passive benchmark index (e.g., equities, cryptocurrencies, bonds, money market investments, seed investments, real estate investments, robo-advisory investments, venture capital investments, private equity investments, and mutual funds & ETFs according to their management approach). Passive investments refer to ETFs and mutual funds according to their management approach. |
| Total expense risk | Excludes positions if they are not ETFs or mutual funds, as TER data only exists for these types of investments. |

In this section, we examine investment risks where these exclusion criteria could lead to biased result due to varying portfolio sample sizes across conditions. We control for these differences.

## Study 1

A single Copilot portfolio gets excluded due to it consisting of positions without a valid ticker symbol (i.e., only savings).

*Geographical Cluster Risk.* We find that the geographical cluster risk effects are not driven by differences in sample size (χ*^2^_ChatGPT-Copilot_*(1, N=179) = 0, *p* > .05; χ*^2^_Copilot-Gemini_*(1, N=179) = 0, *p* > .05) and do not lead to significant median geographical cluster risk differences across conditions (U_ChatGPT-Copilot_ = 3845; U_Copilot-Gemini_ = 4538; U_ChatGPT-Gemini_ = 4132.5; all *p* > .05).

*Sector Cluster Risk.* Analogous to the geographical cluster risk, we find that effects in sector cluster risk cannot be explained by differences in sample size (χ*^2^_ChatGPT-Copilot_*(1, N=179) = 0, *p* > .05; χ*^2^_Copilot-Gemini_*(1, N=179) = 0, *p* > .05) and do not lead to different effects across the median sector cluster risk of conditions (U_ChatGPT-Copilot_ = 3556.5; U_Copilot-Gemini_ = 4523; U_ChatGPT-Gemini_ = 3730; all *p* > .05)

*Total Expense Risk.* Several portfolios were excluded from the total expense risk analysis due to their lack of allocation to ETFs or mutual funds. We find that the difference in sample sizes could potentially influence the effects observed (χ*^2^_ChatGPT-Copilot_*(1, N=154) = 12.99, *p* < .001; χ*^2^_Copilot-Gemini_*(1, N=156) = 17.36, *p* < .001; χ*^2^_ChatGPT-Gemini_*(1, N=174) = .17, *p* > .05). Additionally, our findings indicate that the median TER of both Copilot and Gemini is not significantly different from the benchmark and only ChatGPT’s median TER is above the benchmark TER (U_ChatGPT-Copilot_ = 3915.5, *p* < .001; U_Copilot-Gemini_ = 3576, *p* < .05; U_ChatGPT-Gemini_ = 5369.5, *p* < .001; Z_ChatGPT-Benchmark_ = 616, *p* < .001; Z_Copilot-Benchmark_ = 1112.5, *p* > .05; Z_Gemini-Benchmark_ = 1654, *p* > .05).

## Study 2a

*Total Expense Risk.* In the control prompt condition two portfolios and in the debiased prompt condition 13 portfolios are excluded due to their lack of allocation to ETFs or mutual funds. Analogous to the previous studies we find that TER effects might have been influenced by these different sample sizes (χ*^2^*(1, N=163)= 4.16; *p* < .05). However, we find that debiasing techniques had no effect on the median TER compared to the control prompt condition and remained higher than the benchmark TER (U_control-goal_ = 3502.5, *p* > .05; Z_control-benchmark_ = 742; Z_goal-benchmark_ = 747; both *p* < .001).

## Study 2b

*Total Expense Risk.* In the control prompt condition six portfolios were excluded due to their lack of allocation to ETFs or mutual funds. We observe that TER effects might be influenced by different sample sizes across conditions (χ*^2^*(1, N=174)= 4.31; *p* < .05). But we find that debiasing techniques had no effect on the median TER when compared to the control condition and remained higher than our benchmark TER (U_control-debiased_ = 4280, *p* > .05; Z_control-benchmark_ = 601; Z_debiased-benchmark_ = 856.5; both *p* < .001).

## Study 3

*Total Expense Risk.* In Study 3 nine portfolios were excluded from the control prompt condition and one portfolio from the goal prompt condition for this analysis due to their lack of allocation to ETFs or mutual funds. We find that these differences in sample sizes could potentially drive TER effects (χ*^2^*(1, N=170)= 5.19; *p* < .05). However, the median TER difference between the social responsibility goal formulation and control condition and the respective contrast to the benchmark were significantly different (U_control-goal_ = 1562.5; Z_control-benchmark_ = 615; Z_goal-benchmark_ = 0; all *p* < .001).
