# Supplementary material for: Biased echoes: Large language models reinforce investment biases and increase portfolio risks of private investors
Source: PLoS One. 2025 Jun 27;20(6):e0325459. doi: 10.1371/journal.pone.0325459 (PMC12204588; doi:10.1371/journal.pone.0325459)
Supplement: Attachment — Submitted filename: PONE-D-24-45837_ReviewerX_General-Comments.pdf [file pone.0325459.s005.pdf]

Reviewer #X

1. Foreword

This research investigates the advice given by generative AI (henceforth, GenAI) in investment strategies for private investors. Three GenAIs representing the most common GenAIs used by the general public were used. The prompts to generate the AI advice included variables such as the investor's age and willingness to take risks in their investments. Contrasting the investment advice offered by a trusted investment source, it was found that the advice given by GenAIs showed typical biases in finance and investment. More specifically, GenAI advice tended to propose overly risky investments, i.e. investments with little geographical diversity (i.e. mainly in the US) and sector diversity (generally in one sector at a time), investments linked to recent trends (last 3 months with the risk of buying high and selling low), active investment allocations (risk of high transaction costs and underperformance) and investments with high management and operating costs measured by the Total Expense Ratio (or TER; where preference should be given to low TERs, i.e. lower fees). It was also observed that, despite additional statements in prompts calling for varied investments (Study 2a; narrow debiasing), investments avoiding typical investment biases such as lack of diversity, clusters or active management (Study 2b; broad debiasing) or specific investment goals (Study 3), these statements only mitigated these risks without cancelling them out.

This research addresses a crucial theme regarding the expansion and increasing accessibility of generative AI to the general public. Indeed, it is clear that the performance of these tools is sufficiently convincing for an untrained private investor to be attracted to using them for monetary investments. Nevertheless, as the research rightly points out, the lay investor can risk financial losses without training or a critical eye regarding these pieces of advice. The IT resources used for testing these effects are of outstanding quality. They are readily reproducible (obviously, within the limits that GenAIs never generate strictly identical outputs) and adaptable for further research using the same tools. While it is a criterion for publication in PONE, the availability of computing and analytical resources is a significant advantage for research in this area and for furthering this research.

While the quality of the tools and methods used to achieve these results is indisputable, I have significant concerns regarding the presentation format, which needs substantial improvements. The article's deviation from the typical structure of experimental studies may hinder comprehension. These formatting issues do little to promote its qualities. The theoretical framework is too brief. The methodology lacks several essential components, such as the specifics of the analysis performed. The discussion does not address the relationship between this research and prior studies, as well as its limitations and further research directions [after second reading: I realise that all this is in the Supplementary Information and deserves to be in the article's main body].

2a. Introduction – General comments

To begin with, the introduction is too brief for a topic as crucial as the role of AI and the risks associated with trusting potentially risky financial investment advice. The research establishes part of the territory in the first paragraph (26 – 45) by explaining the importance of the study: three articles highlight the rising application of GenAI in financial investments by private investors (i.e., 1–3), while only one study provides concrete statistics related to the population in the UK (i.e., 4). Insufficient studies are presented, especially since they address the issues arising from the increasing reliance on GenAI. Moreover, while the literature may, let's suppose, be scarce when it comes to the use of GenAI for financial advice, there might be relevant studies in other domains where overreliance can be detrimental and problematic. For instance, overreliance can have adverse effects in fields such as professional relationships, academic advice, etc.

The second paragraph (47 – 58) sets out in more detail how GenAIs are prone to various biases, with several scientific articles supporting the point. However, the second paragraph ends by already presenting the research hypotheses and predictions. Indeed, the importance of research is established, and we have learned that more and more private investors are using GenAI advice and that GenAIs have significant biases. However, the paragraph does not establish the niche: we know neither whether there are counter-arguments to the use of GenAI for financial investments (i.e. that there may be benefits to its use) nor whether there is a gap in this issue, nor does it present questions that current research has not yet been able to answer. This paper does not show any limitations of previous research, how it can be developed further, or what it has missed. All these points are aimed at occupying the niche and giving a solution. Finally, the third paragraph (59 – 67) presents the results obtained right before detailing the method, somewhat echoing what was stated in the abstract. A brief overview of the 'present study' would have been appreciated instead.

Finally, the introduction uses key terms yet fails to clarify or define them in detail. While finance experts may easily understand these terms as they have the necessary background, the scope of this research goes beyond this strict area. It should be accessible to individuals from other fields and, ideally, the general public. This research highlights the dangers of non-experts' over-reliance on GenAI, non-experts who are not aware of those risks. Unfortunately, this article's target audience may struggle to appreciate its implications fully. However, I must acknowledge that these terms are in Table 1, which contains nearly sufficient information to address and understand them. This table, only found in the middle of the Method section, summarises the measures, their definitions, their risks, and the calculations used for their accurate measurement. This table summarises definitions that should have been extensively defined beforehand, i.e., at the beginning of the theoretical background.

2b. Introduction – Small comments

l.48	Even if it is intuitive, is there any source that attests to this claim?
l.43 – 45	These terms should have been defined beforehand to thoroughly understand their implications for the research. There is a risk of back-and-forth-reading between paragraphs.

3a. Methods – General comments

In the beginning, I thought the mentioned factors of the risk tendency, the age, and the GenAI were conditions whose levels would be compared for each study in a comprehensive analysis, as it would have been done in a repeated-measures ANOVA or MANOVA (or linear mixed models with iterations as random effects). As mentioned in lines 72 to 76, we have the impression that a comparison between older and younger people would be made in the same manner as a comparison between the different levels of risk-taking (where higher risk-taking could supposedly lead to more significant biases), or even which GenAI would cause the riskier investments.

No comparison between these levels is made in the Results section of the primary document. We then understand in the Results section that these variations in the prompts are used to give a more representative sample of prompts possibly used by private investors and that these are repeated over ten iterations each, i.e., 27 cross-conditions by 10 iterations for 270 occurrences in total. These iterations are presented as a factor in the experimental design: if it were the case, the levels of this factor would need to undergo pairwise comparisons (e.g., iteration 1 to iteration 2, iteration 1 to iteration 3, etc.). I would recommend presenting the interactions strictly *as iterations* of the three previous variables, not *as a factor* per se.

Nevertheless, these comparisons between risk-tendency, age, and GenAI levels are carried out in the Supplementary Information (SI) but should be presented in the manuscript. Indeed, the SI has enormous potential for analyses that deserve to be presented in the manuscript itself. Moreover, SI should be mentioned less in the manuscript. The manuscript should stand on its own as *the* article, so we do not have to read/refer many times to the SI.

Finally, as we reach the conclusion of the section, there is no presentation of the analyses carried out (it seems there are several, in fact): which tests are used? how many? each measure one by one (several ANOVAs at the risk of increasing likelihood of alpha error)? a single and comprehensive analysis (e.g., MANOVA with 5 dependent variables)? which comparisons are carried out (Tukey HSD, LSD, Bonferroni,…)?

3b. Methods – Small comments

l.74-76	It is not clear who should invest in less risky assets, the younger or the older investors?
l.104	TER refers to Total Expense Ratio, but the abbreviation was not introduced earlier. The financial term ‘Total Expense Ratio’ is not defined earlier (unless I am mistaken).
l.109 – 110	This builds on my earlier point regarding content that might not be understandable to those unfamiliar with the field. The abbreviation ‘FRED’ is neither introduced nor explained, leaving me – and/or others – unable to grasp the sentence fully.

4a. Results – General comments

In general, the studies’ results are well presented. We know where the comparisons are significant and where GenAI is a problem when compared to the benchmark. The results are also interesting: even with the investment disclaimer avoiding bias (Study 2a, 2b, 3), some of them still stand out. However, I recommend compiling all these descriptive statistics into a summary table for easier readability and to allow you more room to elaborate on each result, such as which sectors or geographical areas are over- or under-represented

I found the paragraph (168 – 177) on portfolio returns intriguing, not merely ‘ancillary,’ as it deserves more attention. This raises an interesting question: despite the biases noted in the literature, do investments made by GenAI still yield returns when compared to those made with trustworthy devices? What about each prompt? Despite the identified biases, could GenAI ultimately have merit? Perhaps only over a span of 3 or 6 months, and less so over 1 or 2 years (to the extent we can project that far back from when the AI outputs were generated)?

4b. Results – Small comments

l.145	Which sectors specifically?
l.163	I am surprised that the benchmark does not suggest *any* actively managed funds or single equities.
l.276<br>l.275 – 280<br>l.281 – 287	The ESG score is not mentioned anywhere before, either in the method or in the foreword to the results, nor is its abbreviation presented (i.e., Environmental, Social and Governance score). For a non-expert, it is impossible to guess that ESG represents a score ranging from 0 to 100, where the higher the score, the better it is, and represents high environmental and ethical performance.

5a. Discussion – General comments

As for the introduction, the discussion is too brief and could benefit from further elaboration. The discussion does not mention the links with existing research that would have been presented in the introduction, what answers it provides, what other research it contradicts or agrees with, or the hypotheses it accepts or rejects. Additionally, the discussion does not mention the limitations of the present methodology; variables deliberately left out that could be explored in future studies. Regarding future studies, what new directions and questions could it answer? On this last point, the SI (section 9; p. 22) does suggest some questions that could be further elaborated in the main article.

While it is observed that GenAI carry significant biases, the research does not enough emphasise the importance and presence of the disclaimers, specifically the caution against over-reliance on the advice given and the necessity to consult a professional in the field. Notably, the disclaimers in section 5 (p. 17) of the SI are over 75% of the output content, which is substantial (which is the result of numerous reports and precautions taken by the companies behind the GenAIs).

5b. Discussion – Small comments

l.310	I have some reservations about the term 'critical guide'. While the present article highlights the biases inherent in GenAI, I agree that it shows in what GenAI can be an unreliable adviser. However, it does not establish a checklist or precise safeguard that investors must follow to use the advice of a GenAI 'in a fully informed manner'. It acknowledge the risks, but does not offer any *proper* guidance.

6. Conclusive comments

My comments might seem a bit critical, but I genuinely believe that the methodology is sound, valid and reliable. As I said, the format conceals the quality of the substance of this research. I was pleasantly impressed by how the methodology allows for reliable comparisons between GenAI and benchmark advice. Furthermore, the calculations for assessing risk levels are reliable and objective, and the sources (e.g., the benchmark itself) are of high quality. Table 1 is excellent and provides a good overview of the key measures of this research.
